# Microfluidic viscometry using magnetically actuated micropost arrays

**DOI:** 10.1371/journal.pone.0200345

**Published:** 2018-07-17

**Authors:** Robert M. Judith, Bethany Lanham, Michael R. Falvo, Richard Superfine

**Affiliations:** Dept. of Physics & Astronomy, University of North Carolina at Chapel Hill, Chapel Hill, NC, United States of America; University of Manchester, UNITED KINGDOM

## Abstract

Here we describe development of a microfluidic viscometer based on arrays of magnetically actuated micro-posts. Quantitative viscosities over a range of three orders of magnitude were determined for samples of less than 20 *μL*. This represents the first demonstration of quantitative viscometry using driven flexible micropost arrays. Critical to the success of our system is a comprehensive analytical model that includes the mechanical and magnetic properties of the actuating posts, the optical readout, and fluid-structure interactions. We found that alterations of the actuator beat shape as parameterized by the dimensionless “sperm number” must be taken into account to determine the fluid properties from the measured actuator dynamics. Beyond our particular system, the model described here can provide dynamics predictions for a broad class of flexible microactuator designs. We also show how the model can guide the design of new arrays that expand the accessible range of measurements.

## Introduction

**Fig 1:**
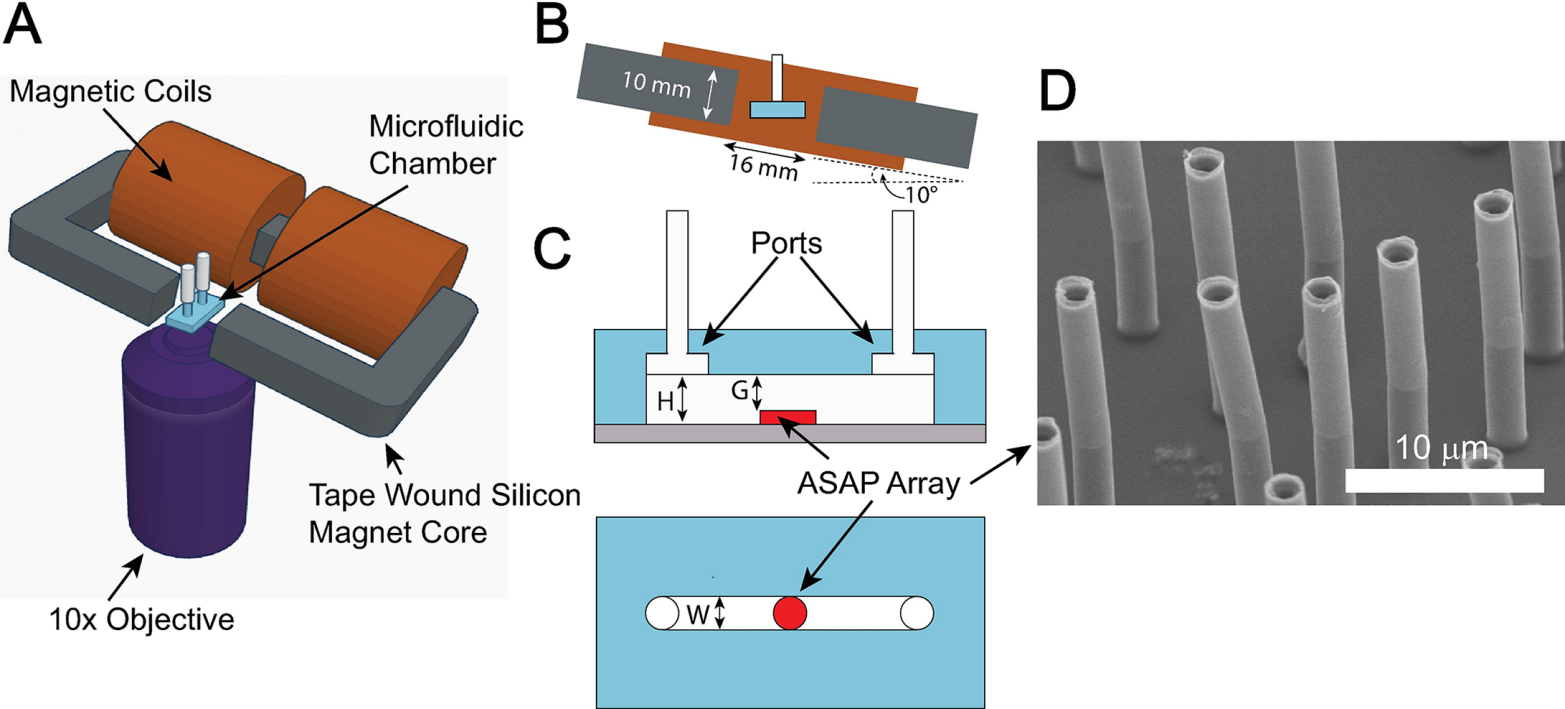
ASAP based Rheometer. (A) Computer generated 3D rendering of the experimental setup. The micro-fluidic channel is placed in between the C-shaped electromagnet core, and is imaged with the microscope objective. The intensity of the transmitted light from the light source is monitored using either a camera or photo diode (not shown). (B) Front view of the microfluidic chamber in between the magnetic poles. The face of the electromagnet is 10 mm X 10 mm and the gap is 16 mm. The magnet is tilted at a 10^o^ angle from the horizontal. (C) A diagram showing the microfluidic chamber. The chamber has a height, *H*, of 250 um. The PDMS base and posts have a total height of 125 um so that the gap height, *G*, between the top of the posts and the ceiling of the channel is 125 um. The channel is 1 mm in width and 16 mm long. (D) SEM of an array of micro-posts. The posts are 2 um in diameter and 23 um tall. Nearest neighbor distance between posts varies between a few um and 15 um.

Rheometry, the measurement of the mechanical properties of fluids, is critically important in areas ranging from basic biology and medicine [1, 2] to industrial contexts such as polymers [3], oil production [4], and food processing [5]. Conventional rheometers use relatively large volumes of material (hundreds of microliters), making studies of precious and novel materials difficult or impractical [6]. In the past decade there has been increasing interest in microfluidics-based rheometers to enable medical diagnostics and basic biological studies on small volume or precious samples [6]. The current push towards lab-on-a-chip technologies that can perform a battery of chemical and rheological tests on a single chip has increased interest in integrating micro-fluidic rheometers into these systems [7–10]. Here we describe a rheometer based on magnetically actuated surface attached post (ASAP) arrays (Fig 1). The post structure consists of a PDMS core that provides mechanical flexibility with a nickel shell on the apical half of the post that drives post motion when exposed to an external magnetic field. We show that quantitative viscosities over a range of three orders of magnitude are determined for samples of less than 20 uL without the need for fluid flow during the measurement. In addition to measuring viscosity, we have previously used these arrays to measure the elastic properties of blood clots [11], and to pump and mix fluids [12, 13].

The success of our ASAP viscometer depends critically on the aid of a comprehensive analytical model we’ve developed that includes the device post material viscoelastic and magnetic properties, the system’s optical readout, and the fluid-structure interactions between the device posts and a viscous analyte. Through use of this model, we find that the beat shape of our actuating posts alters with the relative dominance of viscous drag forces over elastic restoring forces that occurs with an increase of the viscosity and/or beat frequency. For the purposes of this discussion, we use the terminology “beat shape” as distinct from beat amplitude; a change in beat shape denotes a qualitative difference in the beat profile. Taking into account the altered beat shape is essential for predicting actuator behavior across a useful range of viscosity and drive frequency. The model enables successful viscometry, but also provides guidance for future array design that will expand our device’s utility beyond current limitations imposed by our post aspect ratio and the bandwidth of our electromagnet system. The model is more generally applicable to any driven post system where fluid structure interactions affect beat shape, including actuated slender bodies in biological systems.

In the last 10 years, there have been numerous papers on biomimetic cilia arrays and their potential applications for micro-fluidics applications. Like their biological counterparts, artificial cilia arrays have been demonstrated as effective mixers and pumps [12, 14–16], and computational studies have predicted them to be capable of manipulating particle settling [17, 18]. These artificial cilia are typically on the scale of a few microns in diameter and tens of microns tall, and therefore operate in the low Reynolds number regime for typical operational frequencies (< 100 Hz). Arrays have been fabricated using a variety of techniques including using self-assembled micro beads [19], magnetic polymer composites [12], electrostatic and magnetic flaps [20, 21], and core-shell structures [22]. Several numerical and modeling studies have focused on the flow around post arrays [15, 16, 23–28]. Additionally, there is a considerable body of computational work that focuses on the dynamics of biological cilia arrays [29–31]. These computational studies typically either model the fluid dynamics using a prescribed motion of the posts, or are complex numerical simulations that cannot be used to enable experimental measurements of fluid viscosity.

The dynamics of single filament fluid structure interactions have been studied analytically [32–35], and models built on these analytical treatments have been used to describe artificial swimmers and fluid shear sensors [36–38]. In general, the dynamics of these systems depend on a dimensionless parameter known as the “sperm number” [33]. The sperm number, or *Sp*, represents the relative influence of viscous drag forces *between* the surrounding fluid and the actuator with respect to the elastic restoring forces *within* the actuator, and determines the shape of driven post motions:

$$Sp=L{\left(\frac{4\pi \omega \eta }{EI}\right)}^{\frac{1}{4}}$$ (1)

where *L* is the length of the filament, *ω* is the angular frequency of the motion, *η* is the viscosity of the fluid, *E* is the elastic modulus of the filament, and *I* is the second moment of inertia. For *Sp* << 1, the elastic forces dominate, and the deflection of the post is well described by quasi-static Euler beam mechanics. At large sperm numbers, *Sp* >> 1, the viscous forces dominate and the elastic waves formed in the filament become shorter than the filament length. The *Sp* can be thought of as the number of wavelengths that fit within the length of the elastic filament [33]. Changes in beat shape affect fluid flow around the actuator and consequently affect post-post interactions within actuator array settings. Computational studies of the fluid flow generated by actuating post arrays show that the nature of the flow depends strongly on *Sp* [25]. In other studies, simulations show that particle interactions with the post arrays also depend on *Sp* [17, 39, 40].

Multiple lab on a chip rheometer designs have been developed that operate on principles ranging from pressure drop across channels to the onset of turbulence [7, 41, 42]. Microposts employed as fluid motion sensors have been driven by steady and time varying fluid flows, and in high frequency applications near resonance driven by magnetic force at the pillar tip [37, 43, 44]. Artificial cilia arrays however, have not been reported as fluid rheometers. The work presented here represents the first demonstration of a micro-viscometer employing actuated flexible microposts. As previously noted, in prior work, we used our Ni composite arrays as a sensor for the relative elastic properties of human blood clots but did not measure the calibrated absolute viscous or elastic properties of the clot [11].

**Fig 2:**
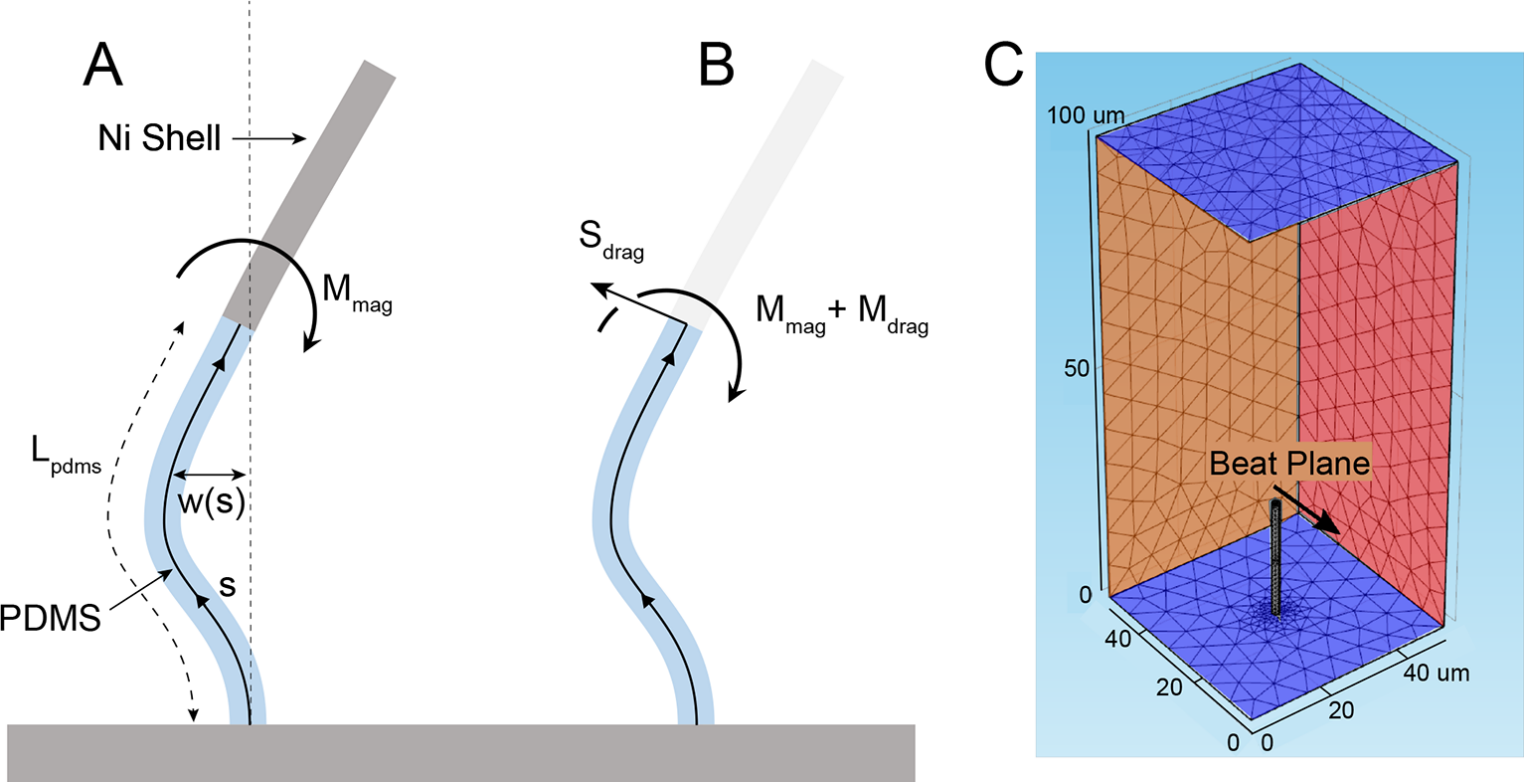
Analytical and computational models. (A) Cartoon of post with analytical model parameters defined. The lower segment (blue) of the post is made of PDMS, and the top (gray) is the Ni shell. The black center line represents the deflection of the beam from the relaxed upright position (parameterized by w(s)). Note that since the Ni segment does not bend, w(s) for the Ni shell for all time points is entirely determined by the position and angle of the PDMS segment at its top end. The applied magnetic field applies a moment to the Ni segment (M_mag_). (B) The analytical model focuses on the PDMS portion of the ASAP and incorporates the magnetic and drag forces experienced by the Ni segment as shear force and moment boundary conditions at the top end of the PDMS segment (S_drag_, M_mag_ and M_drag_). There is an applied moment from the magnetic field, and there are shear and moment drag terms generated by the motion of the Ni portion that are entirely determined by the time dependent position and angle of the top end of the PDMS portion. (C) Rendering from COMSOL showing the mesh and geometry of the finite element model. The model has no slip boundary conditions on the top and bottom, blue; while the left and right sides can have either no-slip or slip boundary conditions, red. The entire post, both the elastic lower portion and the stiffer upper shell, are explicitly modeled. The surfaces perpendicular to the plane of the post motion, orange, have zero pressure boundary condition. Box Dimension: 50 um x 50 um x 100 um.

**Fig 3:**
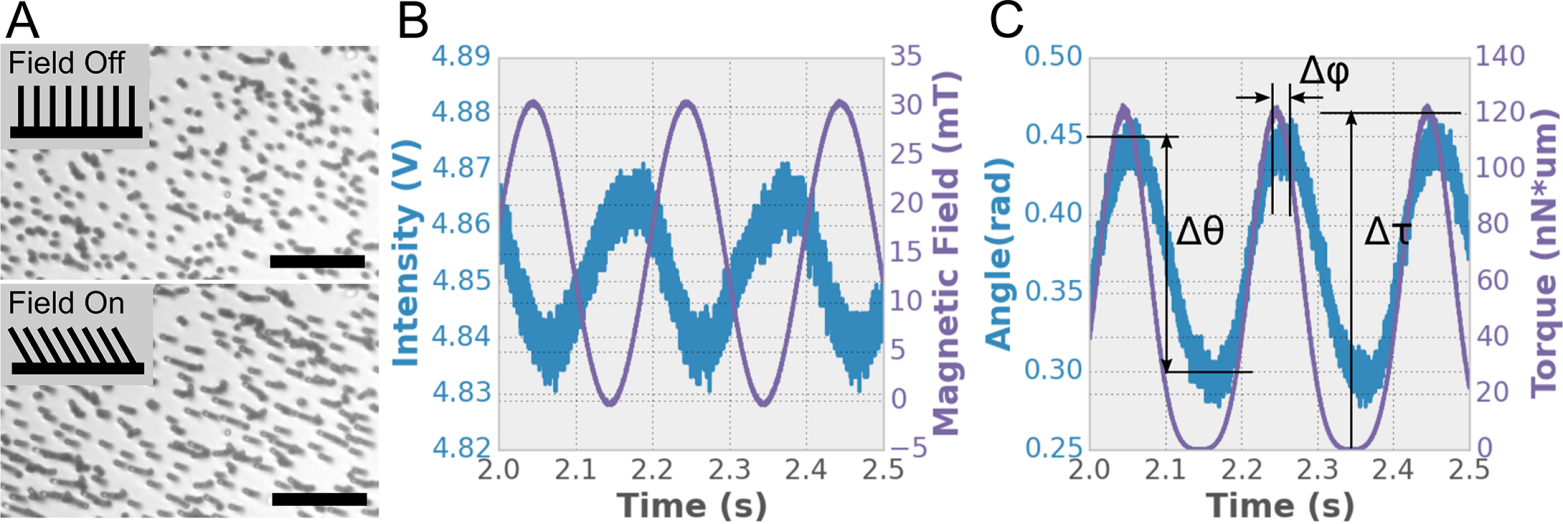
ASAP deflection and phase. (A) Example images of the post array with the field off (top) and on (bottom). Images of this type are used to calibrate the post deflection. Scale bar = 50 um. (B) Raw ASAP results: the magnetic field and the transmitted intensity are measured simultaneously using a DAQ board. The intensity data comes either from integrated whole-frame intensity of video data, or from the photodiode signal (the latter case in this figure). See Materials and Methods. (C) The measured post angle and applied torque are calculated using the tilt and magnetic model explained in detail in [11]. From these results we get the post amplitude, *Δθ*, torque amplitude, *Δ*τ, and the phase, *Δφ*, between the drive signal and the post response.

Here we expand on our analytical model of actuating surface attached posts (ASAP) arrays developed in our previous work [11], to include fluid-structure interactions (Fig 2). From here on, we will refer to our previous analytical model as the “ASAP-1 model”, and the new analytical model including fluid-structure interactions described in this paper as the “ASAP-2 model”. In the experimental system, the bandwidth of the photodiode measurement of the transmitted light through the post array is 10 KHz. This optical measurement is synchronized with the magnetics (driven at 1–30 Hz), enabling precise determination of relative phase of the post response and magnetic driving force (Fig 3). Using the phase data and the ASAP-2 model, we determine the viscosity of the fluid. We show that at low *Sp* (*Sp < 1*), we are able to successfully measure viscosity for fluids ranging from 0.005 to 5 Pa*s. The fluid-structure interaction model that is included in ASAP-2 is necessary to achieve success near *Sp* ~1. For *Sp > 1*, the model fails to predict post array behavior. This suggests a fundamental change in the fluid dynamics at *Sp = 1* that may be due to post-post interactions or post-boundary interactions that are not accounted for in the analytical model. This is consistent with computational studies of post array driven fluid flow at *Sp ~ 1* [25].

## Results

### Analytical model (ASAP-2)

To properly take into account fluid structure interaction, we expanded the model developed by Wiggins [33] to include composite posts. In Wiggins’ model, the flexible filament is treated as a slender beam using Euler Bernoulli beam theory. The forces on the post due to the fluid are modeled as an Oseen drag term [33, 37, 45] (see Supporting Information). In our case, we have a two-component beam instead of a purely elastic beam. The Ni portion of the post is over three orders of magnitude stiffer than the PDMS and will be considered completely rigid. Because the Ni section is effectively rigid for the length scales and drag force magnitudes considered, the motion of the Ni is entirely determined by the motion of the end of the PDMS section (see Fig 2). We therefore need to solve for changes in the shape of the PDMS portion of the post only, while properly accounting for how these changes alter the motion of the distal rigid section of the post. The magnetic torque on the Ni portion which drives the ASAP structure, as well as the drag forces on the Ni portion, are imbedded in the force and torque boundary conditions of the PDMS section (Fig 2). The magnetics and optical read-out components of the ASAP-2 model are the same as within ASAP-1 described previously [11]. Briefly, the tilt model relates the optical intensity readout to the angle of the magnetic portion of the posts through simple geometry (the signal is directly related to the projected “shadow” of the opaque Ni portion of the ASAP posts).

Following Wiggins [32, 33] and others [35, 46] modeling slender bodies in the low Reynold’s number regime where the inertial term and unsteady flow term in the Navier-Stokes equation can be neglected, we use the Stokes equation to describe the hydrodynamics (see Methods and S1 Text). We start with the following differential equation for the deflection of the post from vertical as a function of the arc lengths (see S1 Text for details):

$$EI\;\frac{\partial^{4}w\left(s\right)}{\partial s^{4}}=-\frac{i4\pi \eta \omega }{\ln\left(L_{tot}/2D\right)}w\left(s\right)$$ (2)

$$\frac{s}{L_{pdms}}=\alpha$$ (3)

$$\frac{\partial^{4}w(\alpha )}{\partial \alpha^{4}}=\frac{-i4\pi \eta \omega L_{pdms}^{4}}{EI\;ln(L_{tot}/2D)}w(\alpha )$$ (4)

where *E* is the elastic modulus, *I* is the second moment of inertia, w is the displacement from the relaxed position, s is the distance along the centerline of the post, *η* is the viscosity, *ω* is the angular frequency of the oscillation, *L*_*tot*_ is the total length of the Ni PDMS post, and *D* is the post diameter. The $\ln\;\left(\frac{L_{tot}}{2D}\right)$ term represents the Oseen end correction (see S1 Text), which is why the length considered is the total length of the composite post and not the length of the PDMS section. We nondimensionalize the equations using the parameter $\alpha =\frac{s}{L_{pdms}}$ where *L*_*pdms*_ is the length of the PDMS portion. We can write Eq 4 in terms of *Sp* by substituting in Eq 1.

$$\frac{\partial^{4}w(\alpha )}{\partial \alpha^{4}}=\frac{-i\;Sp^{4}}{ln(L_{tot}/2D)}w(\alpha )$$ (5)

We substitute $k^{4}=\frac{Sp^{4}}{\ln\left(\frac{L_{tot}}{2D}\right)}$ to get the equation into standard form.

$$\begin{array}{c} \frac{\partial^{4}w(\alpha )}{\partial \alpha^{4}}=-ik^{4}w(\alpha ) \end{array}$$ (6)

This equation has a known solution of the form [33]:

$$w(\alpha )=\sum_{j=1}^{4}c_{j}e^{i^{j}z_{o}k\alpha }$$ (7)

$$z_{o}=e^{-\frac{i\pi }{8}}$$ (8)

To solve this equation, we need four boundary conditions. The first two boundary conditions are simply the fixed constraint at the base of the post:

w(0)=0 (9)

$$\frac{\partial w(0)}{\partial \alpha }=0$$ (10)

For the PDMS-Ni boundary, the boundary conditions depend on the motion of the Ni post and the applied magnetic moment. The motion of the post is entirely determined by the motion of the end of the PDMS section. We can then calculate the applied shear and moments to the PDMS end as functions of the motion of the PDMS end:

$$\frac{\partial^{2}w(1)}{\partial^{2}\alpha }=\frac{M_{mag}L_{pdms}^{2}}{EI}+\frac{M_{drag}L_{pdms}^{2}}{EI}$$ (11)

$$\frac{M_{drag}L_{pdms}^{2}}{EI}=\gamma_{1}w(1)+\gamma_{2}\frac{\partial w(1)}{\partial \alpha }$$ (12)

$$\frac{\partial^{3}w(1)}{\partial^{3}\alpha }=\frac{S_{drag}L_{pdms}^{3}}{EI}=\gamma_{3}w(1)+\gamma_{4}\frac{\partial w(1)}{\partial \alpha }$$ (13)

Where *M*_*mag*_ and *M*_*drag*_ are the applied magnetic moment and the moment due to drag forces resolved at the end of the PDMS segment, L_p_ is the length of the PDMS portion, *S*_*drag*_ is the shear applied at the end of the post (see Fig 2), and *γ*_1,2,3,4_ are constants that represent the drag terms on the Ni portion of the post that contribute to the moment (*γ*_1_,*γ*_2_) and shear (*γ*_3_,*γ*_4_). It is straight forward to calculate *γ*_1,2,3,4_, see (S1 Text). It is then a matter of solving the system of four linear equations. The response of the posts is entirely determined by two numbers, the applied moment M, and the parameter k.

It is important to note that for our system *Sp* is dependent on the length of the PDMS portion of the post. This is because *Sp* represents the ratio of the elastic forces to the viscous, and can be thought of as scaled persistence length of the flexible region [33]. The Ni portion of the post is represented in the model entirely by the boundary conditions to the flexible portion of the post. The length used for the end correction term is, however, the total length of the core shell structure, as there are no end effects at the PDMS Ni boundary for fluid flow.

The ASAP-2 model reveals two important factors about our composite ASAP posts. First, the applied moment from the magnetic system factors out of the equation. This means that the beat shape is independent of the applied moment, and that the applied field simply determines the amplitude of the beat shape. Second, the beat shape depends entirely on the ratio of the length of the Ni to PDMS portion of the post and *Sp*. Intuitively this makes sense. The Ni/PDMS length ratio is related to the ratio between the drag forces on the stiff segment to the drag and elastic forces on the flexible segment, and *Sp* represents the ratio of the viscous forces to elastic forces in the system. These two ratios represent the relative scale of all the elastic and drag forces on the composite post.

The ASAP-2 model reveals the qualitative change in beat shape for varying *Sp* (see S1 Fig). At low sperm numbers, *Sp* << 1, the beat shape is symmetrical in time. As *Sp* increases, asymmetry in the beat shape begins to develop on the forward and reverse trajectory. At *Sp = 2*, a clear asymmetry of the beat shapes develops. One interpretation of *Sp* can be found in the wave length of the deflections along the post as compared to the length of the post [33]. At larger values of *Sp*, multiple wavelengths develop in the flexible region of the post (S1 Fig). The number of wavelengths along the post is proportional to *Sp*. ASAP-2 provides predictions for the phase between the driving force and the post response, as a function of sperm number and ASAP geometry (See S2 Fig). As the length of the Ni increases, the Ni drag dominates and ASAP-2 approaches the simpler ASAP-1 model (dashed black line in S2 Fig). Eventually at larger *Sp*, extremely large curvature begins to develop, causing the elastic forces to increase relative to the drag forces which lead to a drop in the phase.

The ASAP-2 model assumes that the post deflection is small, which is often true of the oscillations in our experimental system, but the oscillations are about an equilibrium deflection. ASAP-2 also neglects boundary effects from the floor of the PDMS or post-post interactions. To confirm that we get reasonable results given our model’s approximations, we compared our results with a finite element model (FEM) created in COMSOL. The computational model enables us to look at the post motion and flow generated by a single post (or post array) as a function of *Sp* (See S1 & S2 Movies).

**Fig 4:**
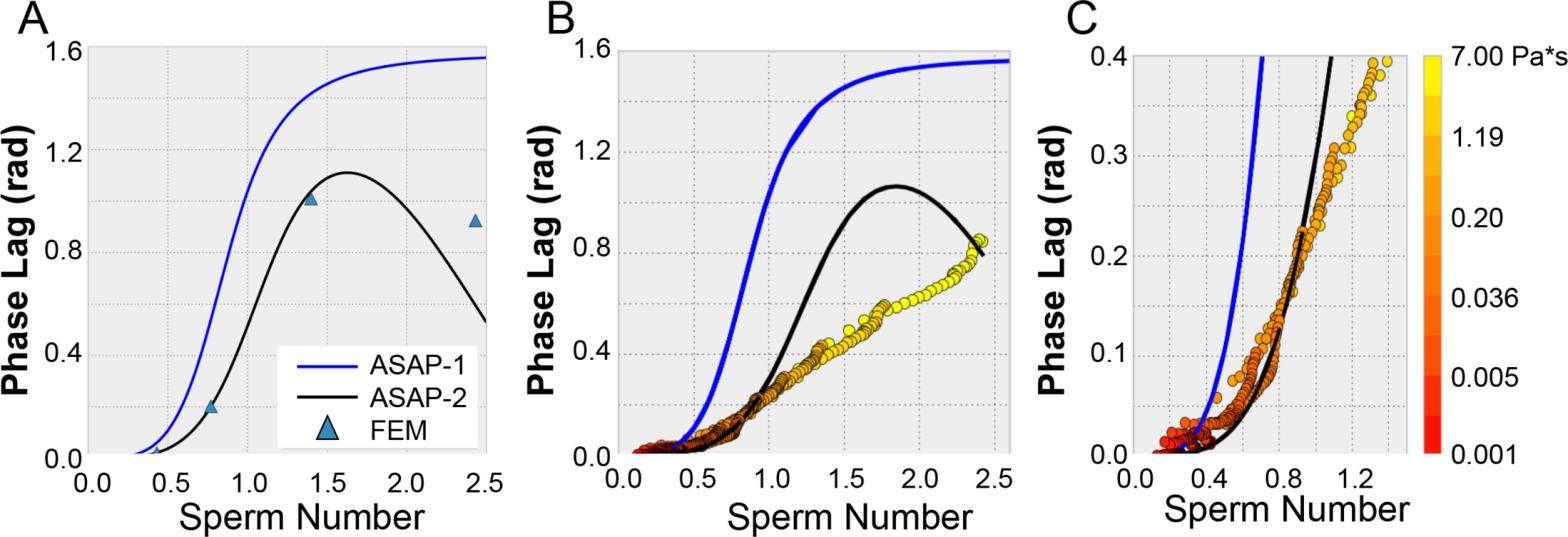
Phase lag of ASAP vs. sperm number. **(**A) Plots of the phase vs *Sp* for the ASAP-1 model, blue, the FEM model, blue triangles, and ASAP-2, black. (B) A plot of the two analytical models, and experimental results as a function of *Sp*. The blue line represents the ASAP-1 model which over estimates the phase at all *Sp* as compared to the ASAP-2 model. Circles represent experimental results at different *Sp*, color indicates the viscosity of the sample (logarithmic scale on right side of figure). Sp was adjusted by both changing the frequency and viscosity of the samples. At *Sp ~ 1*, the ASAP-2 model (black) deviates significantly from the experimental results. Note that the ASAP-2 curves in A and B are slightly different due to differing input shell length parameter to match the FEM and experimental ASAP geometries respectively. (C) Zoomed-in view of B in the low sperm number range.

At low *Sp* there is excellent agreement between the ASAP-2 model and the COMSOL result (Fig 4A and S3 Fig). Our former ASAP-1 model that neglects fluid structure interactions and assumes constant beat shape is depicted by the blue curve in Fig 4A. As the sperm number increases beyond 0.5, the discrepancy between the two analytical models increases. This is particularly obvious in the predicted phase response of the posts (Fig 4A). While both the FEM model and the ASAP-2 model begin decreasing phase as the sperm number increases for large *Sp*, the ASAP-2 model overestimates this change compared to the FEM model. This is reflected in the discrepancies in the beat shape between the FEM and ASAP-2 for large *Sp* as depicted in S3 Movie. This indicates that the ASAP-2 model is either overestimating the elastic component or underestimating the drag component as compared to the FEM model. This may be due to the fact that the FEM model includes the effect of the floor boundary, and the fluid motion, and does not assume that the cross-section of the post remains constant.

### Viscometry results

Fig 4B and 4C depict the phase lag vs *Sp* behavior for our ASAP post system as compared to the ASAP-1 model (blue) and the ASAP-2 (black) (Fig 4C is simply a zoom in of 4B). The experimental ASAP data were collected using fluids of viscosities ranging from 0.005 to 5 Pa*s (color code of data in Fig 4 correspond to fluid viscosity) at drive frequencies between 1 and 30 Hz. For a given trial, the whole range of viscosities and frequency are all performed on the same patch of ASAP (see Materials and Methods) The data do not agree well with the ASAP-1 model for any *Sp* range, whereas they do agree well with the new ASAP-2 model for *Sp* below 1. This suggests that the fluid structure interactions included in the new model and corresponding beat shape alterations are critical to modeling ASAP behavior and achieving successful viscometry.

The ASAP-2 model deviates from the experimental ASAP data above *Sp* ~ 1 as it does with the computational modeling, though the deviation of the ASAP-2 model from experiment occurs at lower *Sp* than from the FEM (Fig 4A). We speculate that this may be due to post-post interaction in the experimental system that are not accounted for in either the ASAP-2 or FEM models.

**Fig 5:**
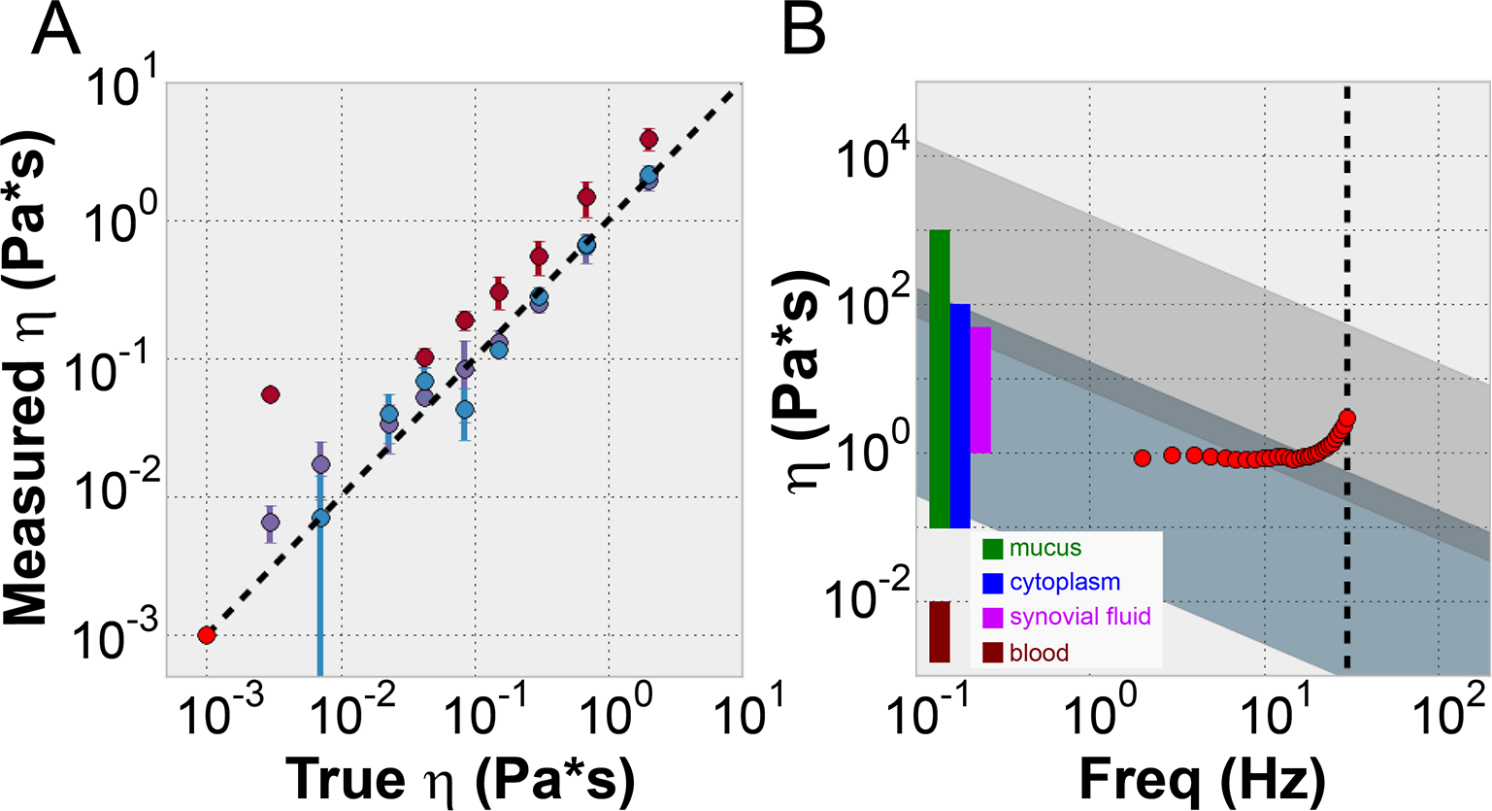
ASAP viscometry. (A) Correlation plot between the measured viscosity and true viscosity of sucrose and Karo solutions. The true viscosity was measured using a cone and plate rheometer, while the measured viscosity was determined from ASAP measurements and the ASAP-2 model using *Sp < 1* conditions. Water was used as the calibration fluid (bright red circle). The colors of the data points represent different post array samples (different experimental trials). Three trials are represented (dark red, purple and blue). (B) Plot showing the experimental range of our current experimental set up (dark blue-gray region), and a proposed alternate design with a 3 um radius and a Ni shell that is ¾ the length of the post (light grey). The red circles represent experimental data taken over a range of frequencies (and a corresponding range of Sp) for a particular viscous sample (nominal 2 Pa*s) using the ASAP system. These viscosity measurements are consistent for *Sp < 1*, but deviate as experimental conditions pass through *Sp = 1*. The black dashed line is the frequency limit of our amplifier. Colored regions on the left represent the low frequency viscosity ranges of biofluids that may be of experimental interest using our ASAP system [47–50].

For a given sample fluid, the ASAP phase was measured at several frequencies between 1 Hz and 30 Hz. The ASAP-2 model was then fit to the experimental phase vs. *Sp* data to acquire a viscosity value. We also note we are constrained by a lower bound *Sp* value as well (*Sp = 0*.*3*) due to the uncertainty the PDMS properties of our system. Fig 5A depicts a correlation plot of ASAP derived viscosity for a range of viscous fluids vs. cone and plate rheometer derived viscosity (referred to here as “true viscosity”). Three different ASAP arrays were used to collect these data (as indicated by color).

## Discussion

### Viscometry analysis

The success of the ASAP-2 model for *Sp* < 1 enables the use of ASAP as a viscometer. We note that this constraint (*Sp* < 1) still provides experimental accessibility to very wide range of fluid viscosities. As Eq 1 indicates, drive frequency (or ASAP geometry) can be adjusted to accommodate for large viscosities to keep *Sp* below 1. Fig 5B depicts the useable range of our ASAP system for reliable viscosity measurement, in viscosity vs. frequency space. The two overlapping diagonal regions (dark grey-blue and light grey) represent *Sp* ranges for particular ASAP-geometries. The grey-blue region represents our current ASAP geometry, while the light-grey represents an alternative geometry that provides access to a complementary region of the phase space. In the right margin of the plot, the viscosity ranges of relevant biofluids are depicted and fall within the usable range of our current ASAP or that of the alternative design. In both cases, the lower boundary of the shaded regions corresponds to *Sp = 0*.*3* and upper boundary corresponds to *Sp = 1*.*0*. The lower boundary (*Sp = 0*.*3*), is due to the confounding effect of the viscoelastic properties of the PDMS which dominate and swamp the signal in low viscosity/low frequency range. At high viscosity/high frequency we enter the large sperm number, *Sp > 1*, regime where the ASAP-2 model begins to show its limitations. The red circles represent viscosity measurements of a nominal 2 Pa*s sample of Karo taken at different ASAP beating frequencies derived using the ASAP-2 model. Below *Sp* = 1, the measured values are accurate and consistent. For higher frequency measurements above *Sp* = 1, the measured viscosity deviates from the true value.

This deviation at *Sp* = 1 is interesting because previous computational studies of driven post arrays indicate that *Sp* = 1 is a critical number. Computational studies have shown that for actuator arrays, the direction of directed flow is predicted to change at *Sp* ~ 1 [25]. It is intriguing that the deviation from experimental results occurs at this number and suggests that the deviation could be caused by a change in the flow dynamics in the chamber that invalidates the single post assumptions implicit in the ASAP-2 model.

It is also possible that the PDMS calibration includes effects of post-post interactions. We calibrated for PDMS properties using the post response in water, which may include the influence of post-post interactions. These interactions may explain why we measured PDMS properties on the high end of the range that is found in the literature [51, 52]. As the posts transition through *Sp = 1*, this calibration may become invalid as the flow in the channel fundamentally changes. The limit of *Sp = 1* for our results limits the experimental range of the system, as shown in Fig 5B. It is possible to design the post arrays in order to bring *Sp* into the valid range for any viscosity. The simplest and most direct way to adjust the post properties is by adjusting the aspect ratio of the PDMS section. *Sp* has two geometric parameters, *L*_*PDMS*_ and the moment area of inertia, *I* (See Eq 1). For a cylinder *I* = $\frac{\pi r^{4}}{4}$, so *Sp* scales linearly with the aspect ratio of the PDMS region, $\frac{L_{PDMS}}{r}$. *Sp* also depends on the frequency. This gives us a simple way to adjust the design of the ASAP post arrays to enable measurement of higher or lower viscosity samples while keeping Sp < 1 (Fig 5B). In the case of design of ASAP, we are limited by geometrical constraints set by ground and lateral collapse [53, 54]. The low driving frequency limit is constrained by phase accuracy as it scales with the number of cycles measured. Our high frequency limit is determined by electromagnetic bandwidth of our magnetics system (200 Hz).

In an array of posts, the fluid flow generated by neighboring posts and its impact on ASAP motion should be considered. Within this work, we show that it is not necessary to take this effect into account to for successful viscometry. However, the FEM model indicates that the posts generate significant fluid flow for their neighbors (S4 Fig). In posts that are separated by 30 *μm*, the fluid velocity drops by less than 20% between the posts. This suggests that post-post interactions in our dense arrays (average spacing 5–6 *μm*) are extremely important. Future work will focus on understanding how these post-post interactions effect the analytical model.

### Other applications

The post arrays have a number of unique advantages. They are easily integrable into micro-fluidic devices, and do not require an externally established flow, thereby reducing the amount of fluid necessary for experiments. Additionally the same ASAP can be used as a mixer or micro-fluidic pump while simultaneously measuring fluid viscosity. For mixtures that change viscosity in time upon mixing, the post arrays could be used as a both a mixer and a measure of the level of mixing. Alternatively, the ASAP elements could monitor the viscosity of the system while acting as a pump in a microfluidic system. We’ve now demonstrated the ASAP system as an effective elastometer [11] and a viscometer, and believe that future improvements to the model and system will allow us to measure the moduli of visco-elastic materials.

ASAP arrays could have potential applications in shear thinning biofluids such as mucus. Given our experimental setup we can approximate the maximum shear that we can produce. If we model the tip of the post as a 2 um sphere we can use the standard equation for the maximum shear rate for a translating sphere [55],

$$|\dot{\gamma }|=\frac{3v_{s}}{{\sqrt{2}r}_{s}}$$ (14)

where *r*_*s*_ is the radius of the sphere, or post in our case, and *v*_*s*_ is the velocity. The max velocity at the tip of the post is:

$$v_{tip}=\theta_{\max}\;L_{post}\omega$$ (15)

where *ω* is the drive frequency of the posts, *θ*_*max*_ is the max post deflection, and *L*_*post*_ is the total length of the post. Substituting Eq 15 into Eq 14, we get the following equation for the tip shear.

$$|\dot{\gamma }|=\frac{3\theta_{max}L_{post}\omega }{{\sqrt{2}r}_{s}}$$ (16)

Assuming a reasonable post amplitude of 10° at 30 Hz, we obtain a shear rate on the order 10^3^ s^-1^, which is large enough to induce shear thinning behavior in biological samples such as mucus and blood, which both have significant shear thinning at shear rates above 10^1^ s^-1^ [56, 57].

For mucus, the ASAP arrays are particularly interesting as they are capable of measuring rheological properties in a way that directly mimics biological cilia in the airway. Rheological measurements of heterogeneous samples such as mucus can vary with the scale of the measurement and the actuation geometry. The current post arrays are not stiff enough to effectively measure the full range of viscosities found in mucus while remaining in the low sperm number regime. This could be adjusted by changing the aspect ratios of the posts. Posts with twice the diameter and 75% of the length of our current ASAP arrays could potentially measure mucus rheology properties while remaining under the *Sp = 1* limit.

## Conclusion

We have demonstrated the first microfluidic viscometer using driven flexible micro-actuators. Our device does not require externally driven flow and is capable of successful measurements of viscosity on sample volumes of 20 *μL* for fluids in the range of 0.005 to 5 Pa*s. We found that alterations of the actuator beat shape as parameterized by the dimensionless “sperm number” must be taken into account to back out fluid properties from the measured actuator dynamics. Critical to the success of our system is a comprehensive analytical model that includes post viscoelasticity, magnetics of the post, optical readout, and fluid structure interactions. The model is applicable beyond our specific system, and can provide dynamics predictions of any flexible microactuator system.

## Materials and methods

### Flow cells

The microfluidic channels were fabricated from PDMS starting with the laser cutting of negatives of the channels out of 250 μm transfer adhesive. The channels were 1 mm in width and 16 mm long. The channel negatives were then attached to the bottom of a 100 mm petri dish. Micro-fluidic ports (Value Plastics, Inc. B000FP9YDO) were then mounted onto the transfer adhesive. 30 ml of PDMS was mixed at a 10:1 PDMS:cross-linker ratio. The PDMS mixture was poured over the channel negatives and allowed to cure at 80 C overnight. The channels were then cut out of the petri dish using a razor. The channels were plasma cleaned for 30 seconds and coated with a thin layer of Norland optical adhesive and sealed onto the ASAP arrays, which were mounted on an 18 x 18 mm number 1 cover slip.

### Electromagnet microscope setup

ASAP actuation was performed with an opto-magnetic system as shown in Fig 1. The posts were imaged using a 10x, 0.3 NA, Plan DL, Nikon objective and a mounted 60 Hz Firefly USB 2.0 camera (FMVU-03MTM-CS). The camera was interchangeable with a Thor Labs amplified photo diode (Thor Labs PDA36A). The diode has a 13 mm^2^ detection area, and is responsive to optical wavelengths from 250–1100 nm. The camera was used to take images of the deflected posts for calibration purposes, while the photo diode was used for measuring the deflection during the oscillatory experiments and the phase relative to the magnetic field. In both cases, the modulating intensity data is coming from an sample area with dozens of ASAPS (full field of view) as depicted in Fig 3A. A collimated 780 nm high intensity LED from Thor labs (Cat# M780L3), with a polycarbonate diffuser, was used as the light source. The noise level of the camera and LED system was less than 0.03% of the average image intensity.

To actuate the posts, an electromagnet consisting of tape wound, silicon steel, C-shaped core with a 10 x 10 mm cross-section and a 16 mm gap was used. Magnetomotive force was produced by two magnet coils, with 680 turns each, connected in parallel for a total of 1,380 turns. The magnetic field varied less than 10% within 3 mm of the gap center, confirmed experimentally and with COMSOL simulations. The electromagnet was driven by a transconductance amplifier. The amplifier and magnets had a combined bandwidth greater than 50Hz. The combination of the amplifier and magnet was able to reproducibly generate a field within the 1% precision of the Gauss probe used to measure the signal.

### Phase measurement

The primary data of the experiment of the experiment are the transmitted intensity through the post arrays as measured by the photo diode, and the magnetic field, which is determined from the current delivered to the silicon tape wound core (Fig 3). The electro magnet has low remanence (< 0:1 mT), and its bandwidth (200 Hz) which is well above the maximum drive frequency (30 Hz). Because we drive the magnet below the saturation threshold, there is a strictly linear relationship between magnetic field and current as confirmed in control experiments. Both the applied magnetic field and the transmitted intensity are converted into a post angle deflection and applied torque using previously developed magnetics and tilt models [11].

From the processed results we can measure the change in post tilt angle amplitude, Δθ, the amplitude of the torque, Δτ, and the phase between the two, Δϕ. We extract these parameters by fitting the tilt angle amplitude and torque data with a sinusoid of the form:

F(t)=Asin(2πft)+Bcos(2πft) (17)

where *f* is the drive frequency, *t* is the time, and *A* and *B* are fitting parameters. Because the magnetic torque is proportional to the square of the magnetic field, there will be higher frequency components in the drive signal and the response. However, by fitting the data with the sine function at only the drive frequency, we suppress the contributions of higher frequencies in the data.

### ASAP rheology protocol

For an ASAP rheology experiment, the posts are subject to an oscillatory magnetic field. The field is an offset sine wave that goes from zero to a maximum and back to zero, and is never negative. In order to calibrate the ASAP post amplitude in different fluids with different indexes of refraction, a steady state experiment is performed at each fluid exchange. A known magnetic field is applied to the post array and the posts are allowed to equilibrate. The change in intensity at the equilibrium position is used to calibrate the post angle, using images taken with the camera prior to the experiment. Because all the materials used are visco-elastic or viscous fluids, over long enough time the post deflection is determined by the magnetic and material properties which remain constant. This allows us to calibrate the post amplitude to intensity changes in fluids with different indices of refraction.

Viscous fluids were loaded into the channel using a syringe pump (Pump Systems Inc. NE-1200). At least 10 mL of fluid was pumped through the channel in between each viscous sample exchange to ensure that all of the previous sample was removed. A frequency sweep was then performed, typically from 1 to 30 Hz in 1 Hz intervals. For a given experiment, the whole procedure (range of viscosities and sweep of frequencies) is performed using the same field of ASAPs.

At each frequency, the phase and amplitude of the post motion and the magnetic field was recorded, using a NI instruments DAQ board, PCI-6713. The magnetic field was read as the current being supplied to the silicon tape wound core, and the intensity is monitored with the Photo Diode. Post amplitudes ranged from 2–8 um as determined from the photodiode signal and our calibration procedure.

Because of the visco-elastic nature of the base of the ASAP posts, a calibration procedure is performed to characterize the complex modulus of the PDMS for every post sample (see S1 Text and S5 Fig). This is essential in order to distinguish the viscous properties of the measured fluid from that of the ASAP posts themselves. The phase lag due to the visco-elastic nature of the PDMS determined for each post array, was subtracted from phase data for viscous samples.

### Viscous samples

Sucrose solutions were made at, 1M, 1.5M, 2M, 2.2M and, 2.4M concentrations. To create viscous samples over the saturation point of sucrose, Karo syrup dilutions were used. K aro was diluted to 95%, 90%, 85%, 80% Karo by weight with water. The viscosity of all samples were measured on a TA Instruments AR-G2 controlled stress, 40mm/1 degree cone rheometer, at 23°C. Sucrose and Karo are Newtonion Fluids [58, 59].

### Computational model

The ASAP fluid system was modeled using the fluid structure interaction model in COMSOL Multiphysics (Version 5.1.0.180). The elastic portion of the post was modeled as an elastic material with an elastic modulus of 1.5 MPa and a Poisson ratio of 0.5. The Ni portion was modeled as an elastic material with an elastic modulus of 1GPa, and a Poisson ratio of 0.5. The magnetic force was included as a force couple applied at the ends of the Ni portion of the post parallel to the end surface, which applied a net torque. The force couple varied sinusoidally according to the function:

$$F(t)=\frac{F}{2}(1-\cos\left(2\pi ft\right))$$ (18)

The force was determined given the known applied field in our magnetic system, the length of the Ni shell and volume of Ni. The calculated force along with the PDMS properties gave deflections in the same range as our experiments.

The mesh used for the finite element model was an automatically generated triangular mesh. The mesh size was reduced until further reductions resulted in less than a 1% change in the model results. The mesh was deformed with the post movement to maintain mesh quality and was smoothed using Hyper-elastic mesh smoothing (see Fig 2).

The fluid structure interaction was solved using the direct fully coupled PARDISO solver in COMSOL. Time stepping was performed using backward differentiation formula (BDF) method with strict steps, and a maximum step size of 0.001 seconds. The boundary conditions on the posts were specified to be fluid structure interactions, with a fixed boundary condition on the base. To allow the post to generate flow, the fluid boundary conditions were set to zero pressure for inlets and outlets on the boundaries perpendicular to the plane of the post motion. The top and bottom of the channel were modeled as no-slip boundaries, while the sides of the channel were modeled as either no slip boundaries or slip boundaries, depending on the goal of the simulation. The slip boundary conditions are that of a single post within an infinite 2D post array with a unit cell equal to the simulated volume, which means post-post interactions are implicitly included. Because of the spatial scale and frequency range of the system, we used the low Reynolds approximation to the Naiver Stokes equations, i.e. Stokes Flow (Re < 0.01 for all experimental conditions).

## Supporting information

S1 Text Hydrodynamics Calcs and PDMS calibration. Analytical treatment of the fluid strucuture interactions, drag model, and the PDMS calibration are discussed.
(PDF)

S1 Fig Beat shape vs. Sp. Examples of the normalized beat shape according to the analytical model at different *Sp* for a composite rod with a 1:1 *L*_*Ni*_ to *L*_*PDMS*_ ratio. The normalized displacement is plotted on the x-axis while the distance along the arc length is plotted on the y axis. As *Sp* increases, the drag becomes more and more dominant in the post motion. One full cycle of the beat is shown going from blue to red in time. As the sperm number increases, the amplitude of the post motion also decreases (see S2 Fig, right panel).
(TIF)

S2 Fig Post phase and amplitude vs. Sp. Plots of ASAP-2 model’s predicted post response to an oscillating magnetic field in phase and normalized post amplitude. The left plot is the phase lag relative to the magnetic driving force plotted against *Sp* for different nickel lengths. The black dashed line represents the predictions of the ASAP-1 model. The right plot shows the normalized post amplitude as a function of *Sp* for the same set of Ni lengths (inset, Ni length in um). The Ni-length is varied for the same total post length (25 um).
(TIF)

S3 Fig ASAP-2 model vs. finite element model. Example beat shapes at three different *Sp*. The normalized displacement, w (x-axis), is plotted against the arc length position along the rod (y-axis). The COMSOL-FEM simulation is in red while the black represents results of the ASAP-2 model. Agreement is excellent at low *Sp*, and begins to deviate at high *Sp*.
(TIF)

S4 Fig Flow generated by beating posts. A plot of the normalized velocity (*U/U*_*max*_) as a function normalized distance from the post tip perpendicular to the beat plane. Post is at center of a box of width W. Different box sizes are shown, 30 *μm*, 40 *μm*, 50 *μm*, and 50 *μm* no-slip boundary condition. The slip boundary condition is equivalent to an array of posts with a spacing equal to the simulation box width. The trend of reduced drop-off in normalized velocity for shorter post-post spacing indicates significant post-post interaction.
(TIF)

S5 Fig PDMS viscoelasticity. Plot of measured elastic modulus of the PDMS posts as a function of the drive frequency. The circles represent the real component of the elastic modulus of the PDMS, while the triangles represent the imaginary part. These curves were calculated using equation S17 using water as the experimental fluid. The three colors represent data from three different ASAP arrays.
(TIF)

S1 Movie FEM simulation of ASAP. FEM simulation of a magnetically actuated ASAP post in a 50 um x 50 um x 100 um volume. Numbers near top and bottom of post indicate deflection in um. Vectors represent local fluid velocity. Boundary Conditions: Top, Bottom, Left-Front, Right-Back are no-slip boundaries. Front-Right and Back-Left are zero pressure boundaries.
(AVI)

S2 Movie Side view of FEM model at low and high Sp. At low SP, beat shape is that of a quasi-static Euler beam. At high Sp beat shape is altered, and elastic waves are apparent. Red vectors indicate local fluid velocity.
(AVI)

S3 Movie Comparison of FEM and ASAP-2 analytical model at Sp > 1. Even at SP as high as 1.4, there is relatively good agreement between the FEM and analytical model for dynamic beat shape. For Sp = 2.44, the discrepancy is more pronounced.
(AVI)
