# Supplementary material for: Microfluidic viscometry using magnetically actuated micropost arrays
Source: PLoS One. 2018 Jul 17;13(7):e0200345. doi: 10.1371/journal.pone.0200345 (PMC6049921; doi:10.1371/journal.pone.0200345)
Supplement: S1 Text — Analytical treatment of the fluid strucuture interactions, drag model, and the PDMS calibration are discussed. (PDF) [file pone.0200345.s001.pdf]

Supporting Information

Microfluidic Viscometry Using Biomimetic Cilia Arrays

Robert M. Judith, Bethany Lanham, Michael R. Falvo, and Richard Superfine

I. Fluid-structure model of posts

To model the fluid-post interaction, we used the low Reynolds number approximation of the Navier-Stokes equations. For a primer on solutions to Navier-Stokes applicable to the flow generated by cilia, we refer the reader to PhD theses on our prior ASAP development that cover the topic in depth [1, 2].

For the model developed in this paper, we focused on the Oseen Tensor and slender body theory for force on a rod, which we used to model the hydrodynamic interaction between the post and the fluid. We started with the Navier-Stokes equation as originally derived by Navier in 1827 for an incompressible Newtonian viscous fluid:

$$\rho\left(\frac{\partial\vec{u}}{\partial t}+\vec{u}\cdot\nabla\vec{u}\right)=-\nabla p+\eta\nabla^2\vec{u}+\vec{F} \quad \text{(S1)}$$

where ρ is the density of the fluid, $\vec{u}$ is the velocity of an infinitesimal fluid unit, p is the pressure, η is the viscosity, and $\vec{F}$ is the applied force per unit volume.

We made two assumptions to simplify the problem. The first assumption was that the instantaneous flow is approximately steady state, removing the $\frac{\partial\vec{u}}{\partial t}$ term. Additionally, because we are in the low Reynolds number regime, $Re=\rho vl/\eta<<1$, inertial effects are not significant so we removed the nonlinear inertial term, $\vec{u}\cdot\nabla\vec{u}$. We are now left with the simplified linear Navier-Stokes equation and the conservation of mass equation:

$$-\nabla p(\vec{r}) + \eta \nabla^2 \vec{u}(\vec{r}) = -\vec{F} \quad \text{(S2)}$$

$$\nabla \cdot \vec{u} = 0 \quad \text{(S3)}$$

It is important to note that these equations are now linear, which means that if two solutions f_1 and f_2 exist then $f_1 + f_2$ is also a solution. This enabled us to model a slender beam, such as our posts, as a series of point forces known as Stokeslets. A Stokeslet is the solution to Eq. S2 for a point force within the fluid, and boundary conditions that vanish at infinity (for more thorough explanation on how to derive flow and pressure fields for a Stokeslet see [3]). Pressure and velocity terms are expressed as:

$$p(\vec{r}) = \frac{\vec{F} \cdot \vec{r}}{4\pi r^3} \quad \text{(S4)}$$

$$\vec{u}(\vec{r}) = \frac{\vec{F}}{8\pi\eta r} \cdot \left(\mathbb{I} + \frac{\vec{r}\vec{r}}{r^2}\right) \quad \text{(S5)}$$

where F is the magnitude of the point force, $\mathbb{I}$ is the identity tensor and $\vec{r}$ is the vector from the origin. $\vec{u}(\vec{r})$ is conventionally known as the Oseen Tensor.

To calculate the force from the fluid on a slender rod, we integrated a series of Stokeslets along the centerline of the body. The Stokeslet force values were chosen so that the fluid velocity at the boundary of the rod equals the velocity of the boundary, i.e. no slip boundary conditions. The force of the Stokeslets was integrated to calculate the force per unit length on the rod (for a detailed derivation of this term, see Nguyen et al. [4]). The end result is that for a slender body, such as our ASAP posts, the force along the posts is modeled as:

$$\text{Oseen Drag} = \frac{4\pi\eta v}{\ln(\frac{L}{2D})} \quad \text{(S6)}$$

where η is the fluid viscosity, v is the velocity of the post, L is the total length of the rod, and D is the post diameter. We will call this the "drag term" which represents the force of the fluid per unit length on the rod.

II. Determining $\gamma_{1,2,3,4}$

To calculate the drag terms, we used the same Oseen drag force for a slender rod, and the displacement of the nickel portion of the rod (Fig. 2). Applying these two terms we obtained the following equation for the drag force per unit length on the nickel rod:

$$D_{ni}(s) = \frac{i4\pi\omega\eta}{\ln(L_{tot}/2D)}\left(w(L_{PDMS}) + (s - L_{PDMS})\frac{\partial w(L_{PDMS})}{\partial s}\right) \quad \text{(S7)}$$

$$D_{ni}(\alpha) = \frac{i4\pi\omega\eta}{\ln(L_{tot}/2D)}\left(w(1) + (\alpha - 1)\frac{\partial w(1)}{\partial \alpha}\right) \quad \text{(S8)}$$

where D_{ni} is the drag per unit length along the nickel potion of the rod, and the substitution $\alpha = s/L_{pdms}$ is used in Eq. S8.

Substituting the drag term into the equation for the moment boundary condition (Eq. 11 in main text), we obtained the following equations.

$$\frac{M_{drag}L_{PDMS}^2}{EI} = \frac{i4\pi\omega\eta L_{PDMS}^2}{EI\ln(L_{tot}/2D)}\int_{L_{PDMS}}^{L_{tot}}(s - L_{PDMS})\left[w(L_{PDMS}) + (s - L_{PDMS})\frac{\partial w(L_{PDMS})}{\partial s}\right]ds \quad \text{(S9)}$$

$$= ik^4 w(1)\int_1^{L_r}(\alpha - 1)d\alpha + ik^4\frac{\partial w(1)}{\partial \alpha}\int_1^{L_r}(\alpha - 1)^2 d\alpha \quad \text{(S10)}$$

Using Eq. 12 (in main text) we obtain γ_1, γ_2 as:

$$\gamma_1 = ik^4\int_1^{L_r}(\alpha - 1)d\alpha \quad \text{(S11)}$$

$$\gamma_2 = ik^4\int_1^{L_r}(\alpha - 1)^2 d\alpha \quad \text{(S12)}$$

Performing the same analysis on the shear boundary conditions (see Eq. 13 in main text):

$$\frac{S_{drag}L_p^3}{EI} = \frac{i4\pi\omega\eta L_{PDMS}^3}{EI\ln(L_{tot}/2D)} \int_{L_{PDMS}}^{L_{tot}} w(s) + (s - L_{PDMS}) \frac{\partial w(L_{PDMS})}{\partial s} ds \tag{S13}$$

$$= ik^4 w(1) \int_1^{L_r} d\,\alpha + ik^4 \frac{\partial w(1)}{\partial \alpha} \int_1^{L_r} (\alpha - 1) d\alpha \tag{S14}$$

Leading to expressions for γ_3, γ_4:

$$\gamma_3 = ik^4 \int_1^{L_r} d\,\alpha \tag{S15}$$

$$\gamma_4 = ik^4 \int_1^{L_r} (\alpha - 1) d\alpha \tag{S16}$$

Now all that is left is to solve the system of four equations for the four unknowns. This is a trivial task but quite messy. Some general comments about the solution: the term M_{mag} factors out of the solution fully, implying that the beat shape is the same regardless of the magnetic field at a given *Sp*, and the amplitude of motion is linearly dependent on the applied field. The solutions have a wavelength along the PDMS portion of the rod proportional to 1/k. As k gets large (large *Sp*) there will be more and more nodes along the PDMS portion of the rod (Fig. S1).

III. PDMS Calibration

PDMS calibration was done in water where the influence of the viscoelastic properties of the PDMS on the driven ASAP motion are orders of magnitude larger than that of the water viscosity for the frequency range measured. The complex elastic modulus of PDMS was calculated by solving for the elastic modulus in Eq. 4, using the phase and amplitude of the posts in water. The first derivative of the solution of the post motion can be related to the post amplitude using the small angle approximation:

$$\frac{\partial \mathrm{w}(s, \tau(\omega), Sp)}{\partial s} \approx \theta(\omega) \tag{S17}$$

where w (s; $\tau(\omega)$, Sp), the post deflection, is the solution to Eq. 4 which depends on the position along the rod s, the applied torque τ, and Sp. Sp depends on the viscosity η, the frequency f, the elastic modulus of the posts E, and the second moment of inertia I. The experimental results gave us $\theta(\omega)$ and $\tau(\omega)$, and I, f, η are all known. We solved for Sp using Eq. S17 for the post arrays in water. PDMS is a partially cross-linked polymer, and therefore has complex viscoelastic properties and is represented as a complex number. Because the viscosity and frequency are determined, we were able to solve for the complex elastic modulus of the PDMS.

The real elastic modulus of the PDMS ranged between 5-10 MPa, while the imaginary component, ranges between 1-3 MPa (Fig. S5). The PDMS properties were not consistent across arrays and therefore each array needed to be calibrated for each experiment. While the PDMS properties measured are reasonable for PDMS [5, 6], they are on the high end of the expected range and are nearly a factor of 10 higher than the PDMS properties measured in previous studies using the DC tilt tests (for tilt test, see [7]) . It is likely that there are post-post interactions, and post-wall interactions that occur between the posts that are being included in the PDMS calibration. The phase lag due to the visco-elastic nature of the PDMS determined for each post array, is subtracted from phase data for viscous samples.

References:

1. Fiser BL. The Design , Fabrication , and Magnetic Actuation of a Microactuator to Accomplish Propulsion and Large Deflection in Viscous and Elastic Environments [Diss.]: University of North Carolina; 2012.
2. Shields A. Biomimetic Cilia Arrays - Fabrication , Magnetic Actuation , and Driven Fluid Transport Phenomena [Diss.]: University of North Carolina; 2010.
3. Lisicki M. Four approaches to hydrodynamic Green's functions -- the Oseen tensors. arXivorg. 2013:5.
4. Nguyen H, Cortez R, Fauci L. Computing Flows Around Microorganisms: Slender-Body Theory and Beyond. The American Mathematical Monthly. 2014;121:810-23. doi: 10.4169/amer.math.monthly.121.09.810.
5. Du P, Cheng C, Lu HB, Zhang X. Investigation of Cellular Contraction Forces in the Frequency Domain Using a PDMS Micropillar-Based Force Transducer. J Microelectromech S. 2013;22(1):44-53. doi: 10.1109/Jmems.2012.2213070. PubMed PMID: WOS:000314726900009.
6. Fuard D, Tzvetkova-Chevolleau T, Decossas S, Tracqui P, Schiavone P. Optimization of poly-di-methyl-siloxane (PDMS) substrates for studying cellular adhesion and motility. Microelectron Eng. 2008;85(5-6):1289-93. doi: 10.1016/j.mee.2008.02.004. PubMed PMID: WOS:000257413400139.
7. Judith RM, Fisher JK, Spero RC, Fiser BL, Turner A, Oberhardt B, et al. Micro-elastometry on whole blood clots using actuated surface-attached posts (ASAPs). Lab Chip. 2015;15:1385-93. doi: 10.1039/C4LC01478B. PubMed PMID: 25592158.
