# Supplementary figures and images for: Microfluidic viscometry using magnetically actuated micropost arrays
Source: PLoS One. 2018 Jul 17;13(7):e0200345. doi: 10.1371/journal.pone.0200345 (PMC6049921; doi:10.1371/journal.pone.0200345)

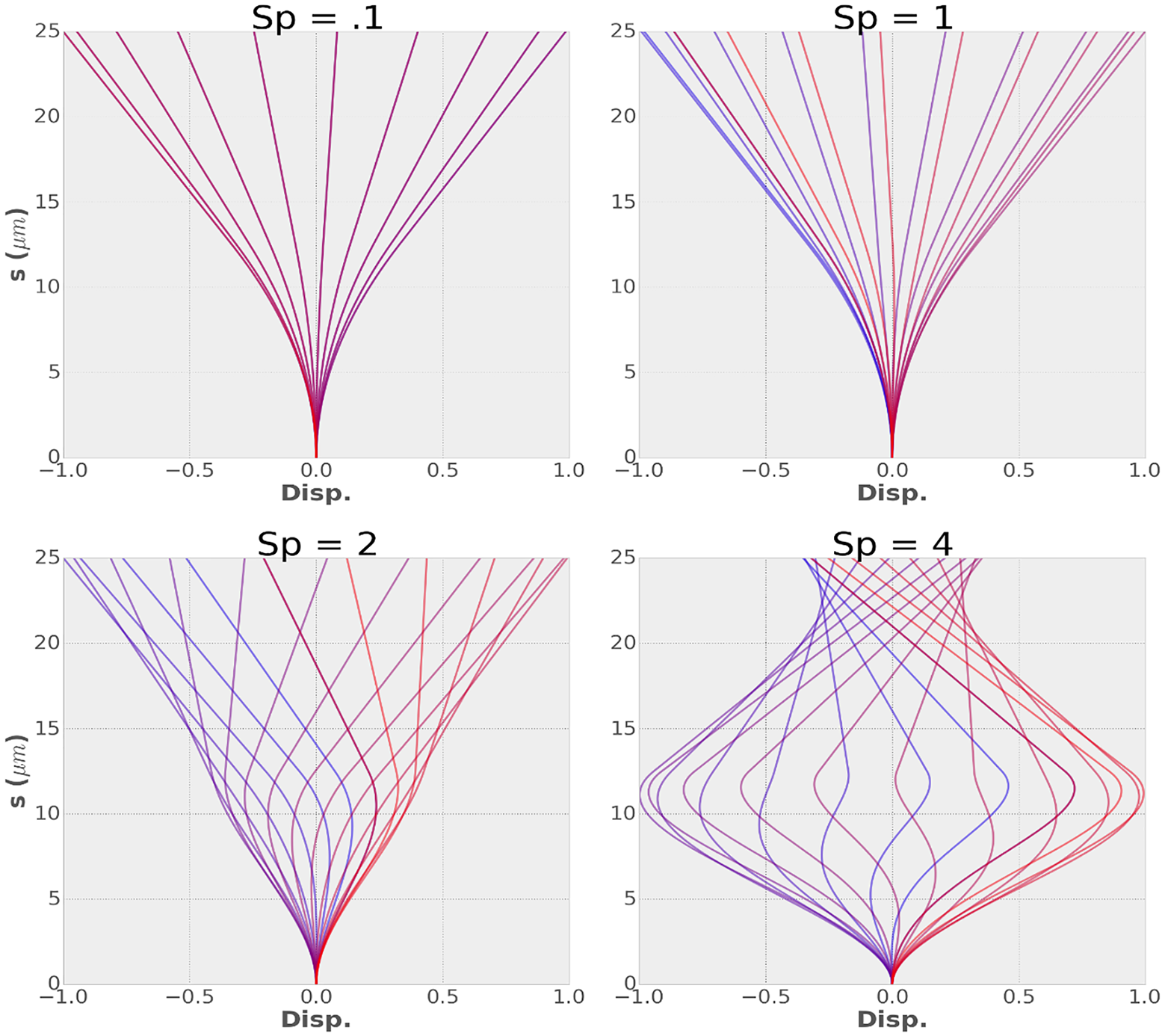

Supplement: S1 Fig — Examples of the normalized beat shape according to the analytical model at different Sp for a composite rod with a 1:1 LNi to LPDMS ratio. The normalized displacement is plotted on the x-axis while the distance along the arc length is plotted on the y axis. As Sp increases, the drag becomes more and more dominant in the post motion. One full cycle of the beat is shown going from blue to red in time. As the sperm number increases, the amplitude of the post motion also decreases (see S2 Fig, right panel). (TIF) [file pone.0200345.s002.tif]

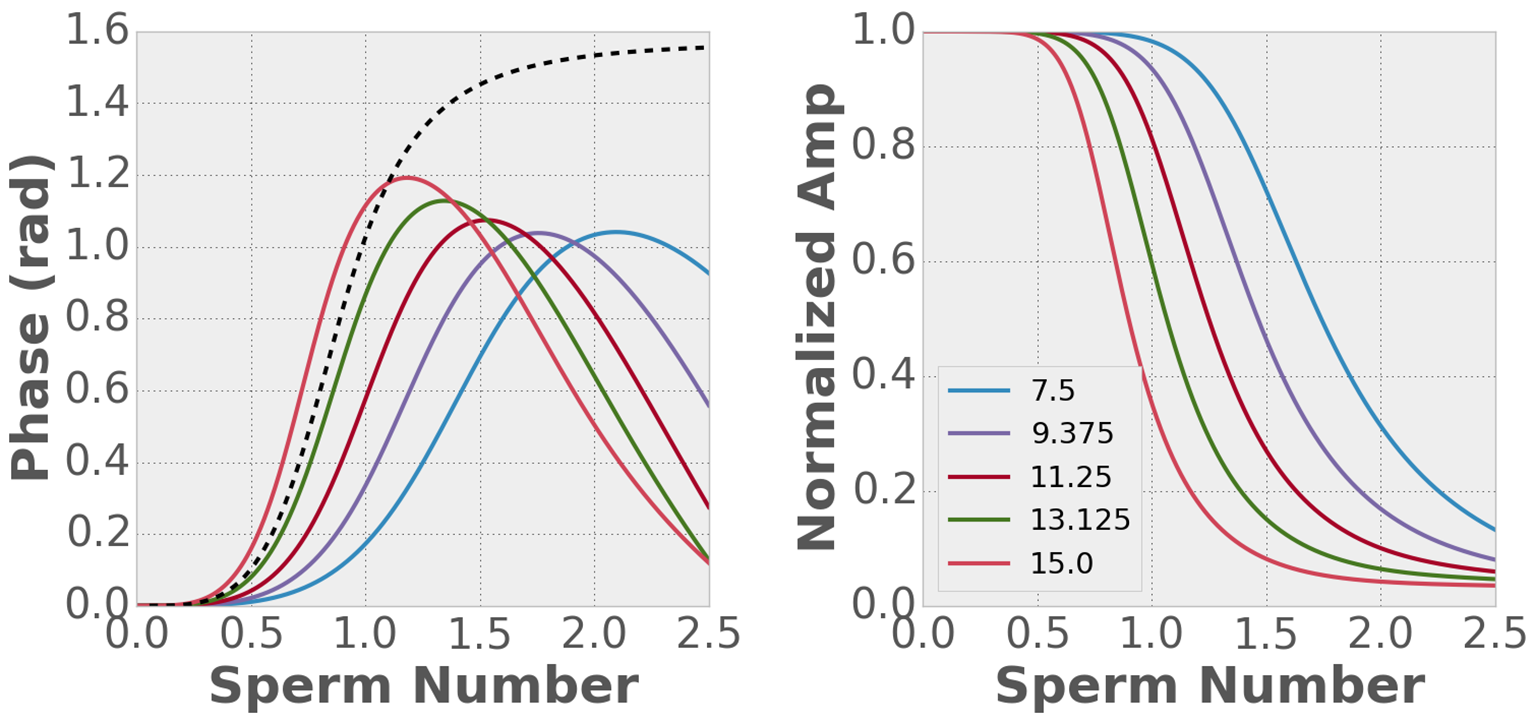

Supplement: S2 Fig — Plots of ASAP-2 model’s predicted post response to an oscillating magnetic field in phase and normalized post amplitude. The left plot is the phase lag relative to the magnetic driving force plotted against Sp for different nickel lengths. The black dashed line represents the predictions of the ASAP-1 model. The right plot shows the normalized post amplitude as a function of Sp for the same set of Ni lengths (inset, Ni length in um). The Ni-length is varied for the same total post length (25 um). (TIF) [file pone.0200345.s003.tif]

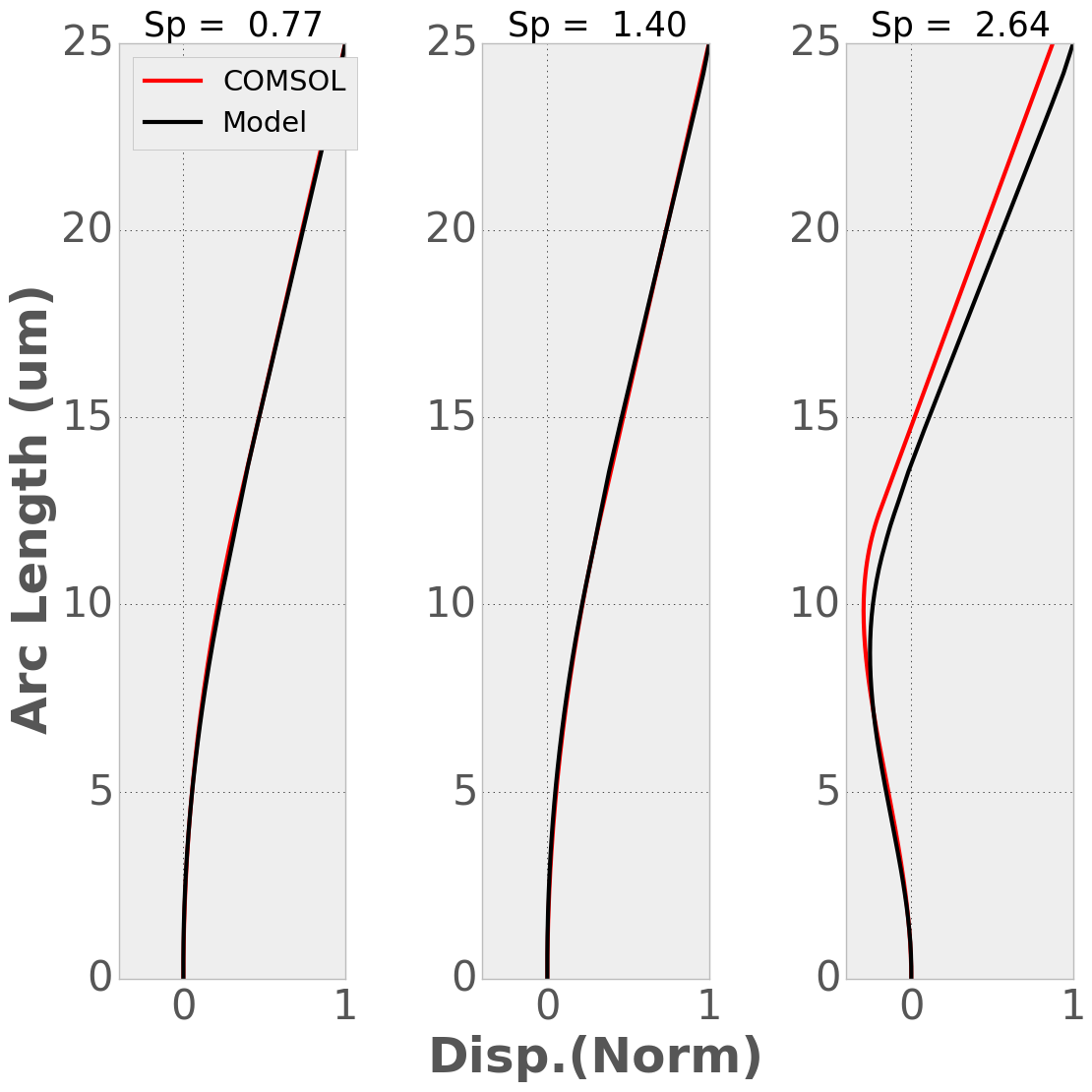

Supplement: S3 Fig — Example beat shapes at three different Sp. The normalized displacement, w (x-axis), is plotted against the arc length position along the rod (y-axis). The COMSOL-FEM simulation is in red while the black represents results of the ASAP-2 model. Agreement is excellent at low Sp, and begins to deviate at high Sp. (TIF) [file pone.0200345.s004.tif]

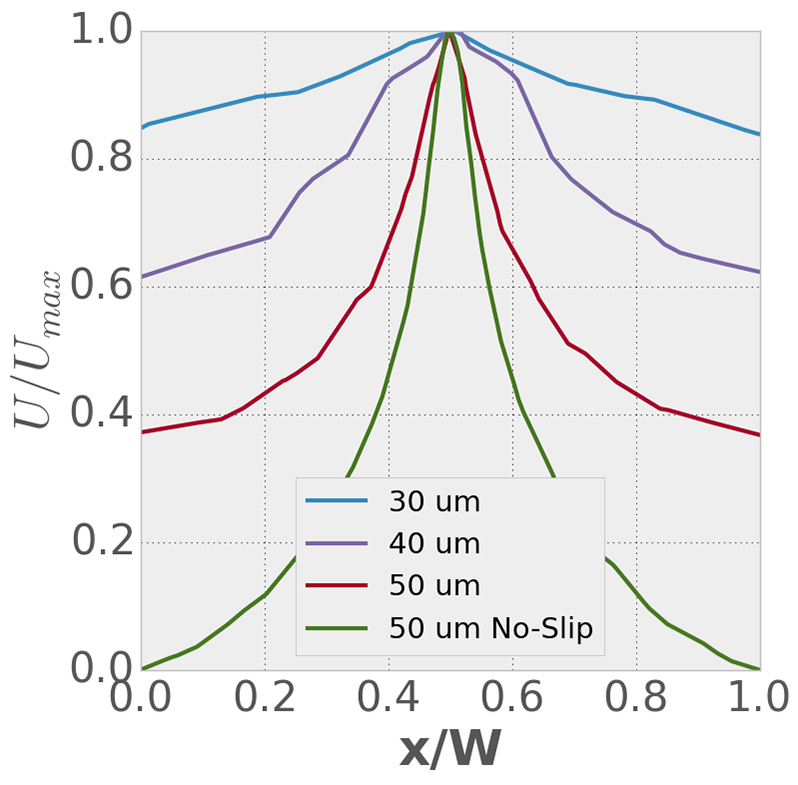

Supplement: S4 Fig — A plot of the normalized velocity (U/Umax) as a function normalized distance from the post tip perpendicular to the beat plane. Post is at center of a box of width W. Different box sizes are shown, 30 μm, 40 μm, 50 μm, and 50 μm no-slip boundary condition. The slip boundary condition is equivalent to an array of posts with a spacing equal to the simulation box width. The trend of reduced drop-off in normalized velocity for shorter post-post spacing indicates significant post-post interaction. (TIF) [file pone.0200345.s005.tif]

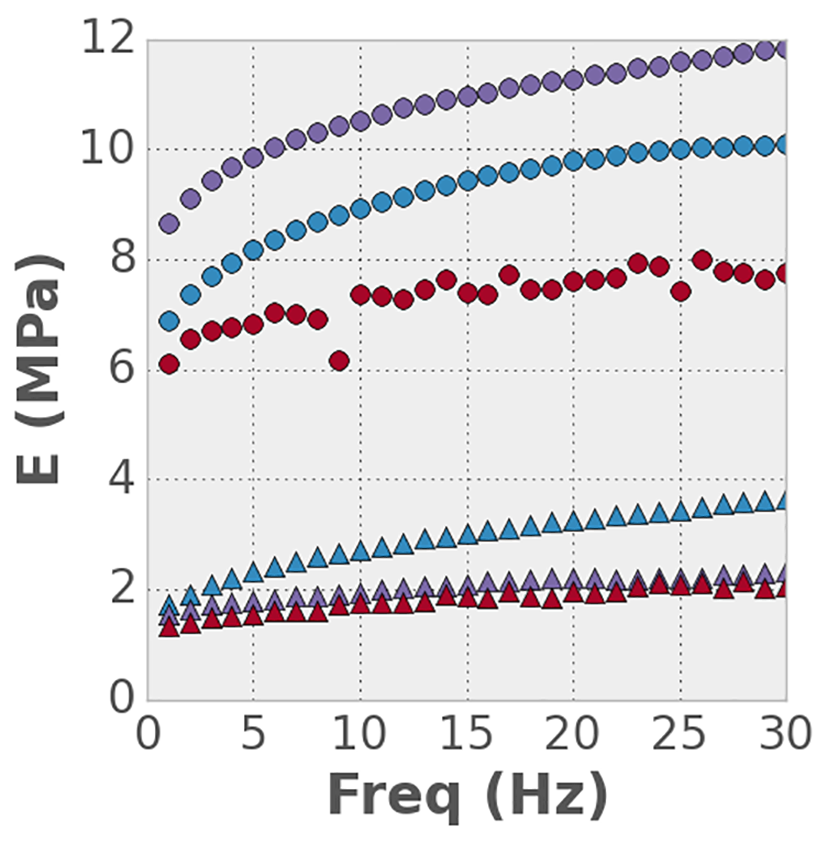

Supplement: S5 Fig — Plot of measured elastic modulus of the PDMS posts as a function of the drive frequency. The circles represent the real component of the elastic modulus of the PDMS, while the triangles represent the imaginary part. These curves were calculated using equation S17 using water as the experimental fluid. The three colors represent data from three different ASAP arrays. (TIF) [file pone.0200345.s006.tif]
